# Preanalytical Workflow Establishment for Reproducible Clinical Blood-Based Infrared Molecular Fingerprinting

**DOI:** 10.1021/acs.analchem.6c01358

**Published:** 2026-06-17

**Authors:** Katharina E. Dietmann, Guanting Guo, Jacqueline Aschauer, Tarek Eissa, Frank Fleischmann, Mihaela Žigman

**Affiliations:** † Department of Experimental Physics−Laser Physics, Ludwig-Maximilians-Universität München, 85748 Garching, Germany; ‡ Laboratory for Attosecond Physics, Max Planck Institute of Quantum Optics, 85748 Garching, Germany; ∇ University Hospital, Ludwig-Maximilians-Universität München, 81377 München, Germany; ¶ School of Life Sciences, Technical University of Munich (TUM), 85354 Freising, Germany; ⊥ Faculty of Medicine, Institute of Pathology, Ludwig-Maximilians-Universität München, 80337 München, Germany

## Abstract

Standardized preanalytical sample handling is a cornerstone of reliable molecular analytics, particularly where subtle multimolecular differences can drive biological interpretation. In different clinical studies, blood-based samples are often processed using varying workflows, and the influence of individual technical parameters remains poorly quantified. In particular, preanalytical differences can limit study comparability and complicate the robust evaluation of the approach for different applications and specific medical questions. Here, we systematically investigate the effects of major preanalytical variables on infrared molecular fingerprinting (IMF) of blood serum and plasma, performed via Fourier transform infrared (FTIR) spectroscopy. We examine the impacts of venous blood draw tube types, tube fill volumes, delay times prior to centrifugation, centrifugation conditions, freezing and thawing procedures, and delay times between final sample preparation and infrared spectroscopy. We found that the type of blood collection tube, the fill volume of the tube, a delay of more than 4 h before centrifugation, more than two freeze–thaw cycles, and delays prior to measurement can result in systematic changes in infrared fingerprints that are detectable through multivariate analysis and machine learning. Importantly, for most conditions, the magnitude of parameter-related spectral variation remains smaller than the intrinsic biological variability across individuals, supporting the feasibility of IMF for realistic clinical settings. The presented results provide actionable guidance and a basis for harmonizing preanalytical workflows, reducing preanalytical confounding in IMF-based biofluid analysis, and improving cross-study comparability, with relevance that may generalize to other molecular profiling technologies, clinical studies, and biobanking.

## Graphical abstract

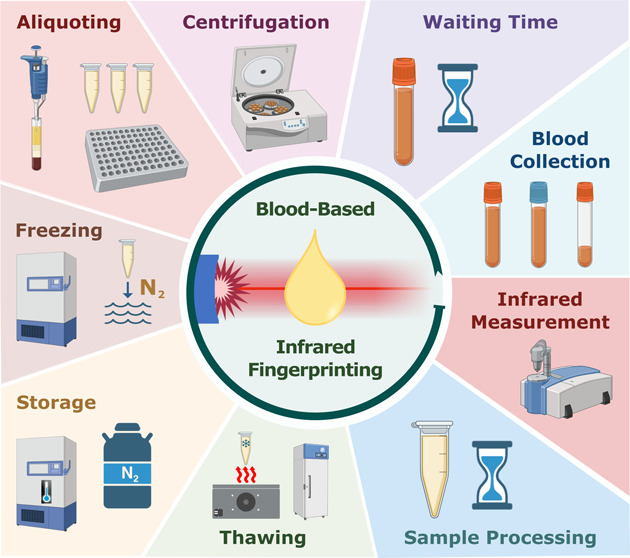

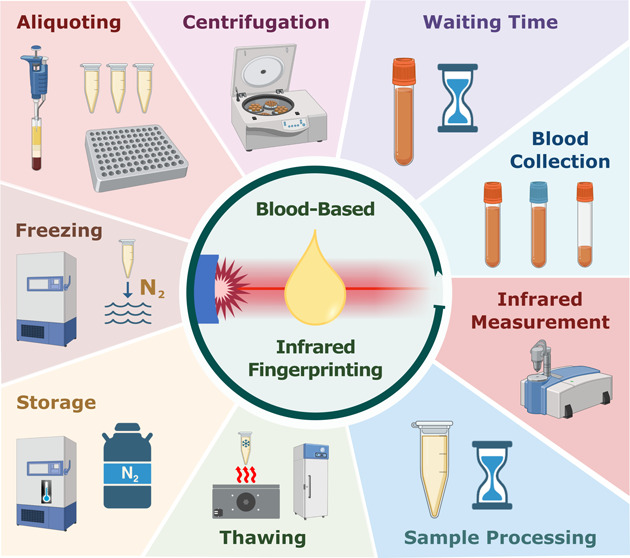

Fourier transform infrared (FTIR) spectroscopy provides a rapid, label-free analytical technique for analyzing the complex multimolecular composition of biological samples such as blood-derived matrices. When combined with multivariate analysis and machine learning, the infrared (IR) spectral molecular fingerprints obtained have been used to detect disease and stratify health states.^1,3,13,25,26,33^ Despite increasing efforts to standardize blood sampling, preanalytical workflows, and measurement procedures, these processes often remain heterogeneous and differ in several aspects across studies, laboratories, and investigations, introducing systematic sources of variability. Sources of variation involve the type of collection tube used, the chemistry of the tube’s additives, draw and fill volumes, delays before centrifugation, centrifugation regimes, freezing and thawing procedures, the history of freeze–thaw cycles, and post-thaw handling prior to measurement. Each of these parameters can, in principle, change the biochemical properties of the sample (e.g., through alterations in molecular conformation, degradation, or changes in hydration state) and thus alter the measured IR spectrum. Even when such preanalytical effects are small in magnitude and may seem negligible when visually examined, these effects can lead to subtle pattern variations that can be picked up by machine learning models. If unrecognized or uncontrolled, such effects can confound downstream analyses, bias outcomes, compromise the robustness and interpretability of downstream analyses, and lead to model failure when independent data is collected.

Preanalytical sample handling parameters have been established as confounders in both mass spectrometric,^7,18,27^ assay-based^9^ and NMR blood-based analyses,^12,16,29^ yet their potential to introduce spectroscopic bias in FTIR-based infrared molecular fingerprinting (IMF) remains unknown. While previous investigations of blood sera with ATR-FTIR were proposed to be robust against common variations in centrifugation and storage conditions (short-term 4 °C storage, long-term −80 °C storage),^4^ the potential for these parameters to introduce subtle biases that could confound machine learning models, particularly when evaluated across different matrices, remains to be fully quantified. Beyond clinical handling, ambient conditions during sample preparation have also been identified as critical confounders in FTIR spectroscopy of blood plasma and serum, for instance, fluctuations in relative humidity can introduce variance potentially masking biological signatures.^32^ These concerns have been recently underscored through proteomics showing that cellular contamination from platelets and erythrocytes, due to variations in preprocessing protocols, can artificially inflate data depth and lead to the misidentification of contaminants as disease biomarkers.^17^ We hypothesized that analogous biases may systematically distort infrared spectroscopic signatures of blood-derived samples. To test it empirically, we conducted a systematic evaluation of several major clinical and preanalytical parameters in the sample collection and processing chain, quantifying their impact on FTIR-based IMF of human blood plasma and serum. Using venous blood sampling, we evaluated matrix-dependent IR fingerprinting (plasma, serum) and quantified the effects of collection tube specifications and incomplete filling, processing delays, centrifugation conditions, freezing and thawing, repeated freeze–thaw cycles, and post-thaw time prior to measurement. To contextualize the magnitude of these preanalytical effects relative to biological variability and heterogeneity, we benchmarked spectral changes introduced by clinical sampling and handling variables against within-person and between-person biological variability. This led us to identify the most influential parameters, guiding the prioritization of standardization measures.

Together, this work provides an evidence-based framework for establishing harmonized IMF workflows to improve analytical robustness and facilitate cross-study comparability. By systematically evaluating preanalytical sample-handling variables, we derived practical recommendations for standardizing workflow protocols across studies, sampling procedures, and laboratory sample-management schemes. Such standardization and harmonization are critical for translating IMF to large-scale clinical investigations and biobank-level studies and are further applicable to other analytical techniques used for quantitative analysis of human venous blood.

## Experimental Section

### Study Setup and Ethics Approval

To systematically quantify whether and how clinical and preanalytical sample-handling factors, such as the type of blood collection tubes, centrifugation settings, and freezing and thawing processes, influence blood-based infrared molecular fingerprints, we performed FTIR spectroscopic measurements on venous blood-based samples obtained from 18 healthy individuals as well as on quality control pooled human blood serum (QC serum). Participants provided repeated peripheral blood donations under defined collection and processing conditions, and the samples were processed into either plasma or serum. All enrolled individuals provided written informed consent prior to their enrollment. The study was approved by the Ethics Committee of the Ludwig-Maximilians-University (LMU) of Munich (ethics approval no. 20-119), was registered at the German Clinical Trials Register DRKS (ID DRKS00034935), adhered to all relevant ethical guidelines, and was conducted in accordance with Good Clinical Practice (ICH-GCP) and the Declaration of Helsinki.

### Standard Operating Procedure (SOP) for Blood Sampling, Processing, Management, and Storage

Peripheral venous blood was collected from an antecubital vein using sterile 21-gauge Safety-Multifly needles (Sarstedt, Nümbrecht, Germany). Immediately after blood collection and tube filling, all tubes were gently inverted 10 times to enable thorough mixing with the respective additives. Blood serum was collected in S-Monovette Serum Gel CAT (S-CAT) tubes (Sarstedt, Nümbrecht, Germany) with nominal volumes of either 4.9 mL or 7.5 mL. For complete coagulation, all serum tubes were kept upright at room temperature for 30 minutes. Blood plasma was collected in S-Monovette EDTA K3E (S-K3E) tubes (Sarstedt, Nümbrecht, Germany) with nominal volumes of 4.9 mL and 7.5 mL, and in Vacuette K2E and K3E (G-K3E and G-K2E) tubes (Greiner Bio-One, Frickenhausen, Germany) with nominal volumes of 4 mL and 10 mL. Whenever serum and EDTA-plasma were sampled from the same venipuncture, serum was collected first.

As the benchmark reference procedure, samples were centrifuged (Centrifuge 5810R, Eppendorf SE, Hamburg, Germany) at 2000 g for 10 min within 1–2 h after collection. The resulting supernatant was divided into 0.5 mL fractions and transferred into cryotubes with screw caps, then immediately stored at −80 °C. To prepare the samples for FTIR analysis, 0.5 mL aliquots were thawed at 4 °C, briefly vortex-mixed to ensure homogeneity, and centrifuged at 2000 g for 10 min. After centrifugation, the samples were dispensed into 90 μL aliquots in cryotubes, and sealed with a pierceable TPE septum, followed by further storage at −80 °C until FTIR measurements were performed.

### Standardized FTIR Measurements

Prior to FTIR measurements, samples were thawed at 4 °C, briefly vortex-mixed, and centrifuged at 2000 g for 3–5 min. Unless otherwise stated, FTIR measurements were performed after two freeze–thaw cycles. Infrared spectra were recorded using an automated FTIR spectrometer (MIRA Analyzer, Clade GmbH, Esslingen, Germany) with a flow-through transmission cuvette (CaF_2_ windows, effective path length of 8 μm) at a resolution of 4 cm^–1^ over the range of 930–3050 cm^–1^. After each sample measurement, a water reference spectrum was recorded and used to reconstruct the infrared absorption spectra.

Analytical stability and technical measurement variability were monitored using pooled human blood serum (quality control (QC) serum, Biowest, Nuaillé, France), which was measured after every five samples. The measurement cell was automatically cleaned during sample exchange. Samples were measured in randomized order to minimize potential systematic measurement-order effects. A typical measurement batch consisted of 25 samples and 6 QC samples, completed within 1 h and 55 min. During acquisition, samples were kept in the instrument tray at room temperature. After each batch, an extended cleaning procedure was performed according to the manufacturer’s recommendations.

### Testing the Effect of Blood Collection Tube Type and Fill Volume

To quantify the impact of blood collection tube type and fill volume, we compared infrared molecular spectra from samples collected in different plasma and serum tube types with different nominal volumes, as well as from partially and fully filled tubes. Here, we focus on EDTA plasma only and do not study other plasma formulations. For the evaluation of nominal tube volumes, S-Monovette serum and plasma tubes (S-CAT and S-K3E) with nominal volumes of 4.9 mL and 7.5 mL were used. Insufficient tube filling (underfilling) was tested by collecting venous blood in S-Monovette serum and plasma tubes (S-CAT and S-K3E) that were filled to approximately 50% of their nominal tube volume. For the comparison of different tube types, we used S-K3E (4.9 mL), G-K2E (4.0 and 10.0 mL), and G-K3E (4.0 mL) tubes. Following venipuncture, all samples were processed according to the standard operating procedure (SOP) and measured as technical duplicates. The experimental setting is summarized in Table S1.

### Testing the Effect of Centrifugation Conditions

To assess the influence of different centrifugation procedures on IMF, we systematically varied the relative centrifugal force and the centrifugation duration. In addition, we investigated the effect of the time interval between blood draw and centrifugation (precentrifugation delay) by varying the interval from 0.5 h to 6  h, while keeping the reference centrifugation parameters (specified in the SOP) constant. Both investigations were performed using S-Monovette K3-EDTA (S-K3E 4.9 mL) collection tubes. The experimental overview is shown in Table S2.

### Testing the Effect of Freezing and Thawing Parameters

Plasma samples from three participants were collected in S-Monovette K3-EDTA (S-K3E 4.9 mL) tubes and used to investigate the effect of different freezing and thawing conditions on infrared molecular fingerprints. All samples were aliquoted according to the standard operating procedure and analyzed as technical triplicates. All combinations of two freezing and two thawing procedures were tested. Uncontrolled freezing (UF) was performed by placing aliquots directly at – 80 °C for at least 3.5 h. Controlled freezing (CF) was performed using a cold workbench (WB230, Askion, Gera, Germany), which automatically froze samples to – 80 °C within 15 min using a temperature gradient in the headspace above liquid nitrogen. Uncontrolled thawing (UT) was performed by thawing samples at 4 °C for at least 80 min, whereas controlled thawing (CT) was performed using an automated thawing device (Arizona, SPL Guard, Drenthe, The Netherlands), in which samples are thawed up to 4 °C in a controlled air stream.

Using a sample set from five individuals, we investigated the effect of repeated freeze–thaw cycles on infrared molecular fingerprints by subjecting aliquots to up to eight consecutive freeze–thaw cycles under UF and UT conditions, with seven technical replicates per participant. The experimental organization is shown in Table S3.

We further investigated the impact of storage temperature and duration on IMF. To test this, we exposed over 90 QC serum samples to different storage temperatures after aliquoting. Samples were stored overnight at – 80 °C and for 1 to 8 weeks at – 20 °C, 4 °C, and room temperature (RT), then compared to the – 80 °C reference samples. To identify temperature-dependent changes, we projected each spectrum into a two-component Principal Component Analysis (PCA) score space and calculated the Euclidean distance from the – 80 °C reference centroid. Group distances were analyzed using two-sided Mann–Whitney U tests, with p-values corrected for multiple comparisons via the Bonferroni method. Results are shown in Figure S6 and Table S5.

### Testing the Effect of Delays before Measurement

To evaluate the impact of post-thaw standing times on infrared molecular fingerprints, defined as the interval between thawing (according to SOP) and FTIR measurement, QC serum was repeatedly measured over 3 h. Infrared spectra were acquired at 3.5 min intervals and grouped into four consecutive time windows spanning 0 to 3 h after thawing (post-thaw). The experimental overview is shown in Table S4.

### Quantification and Statistical Analysis

Infrared spectra were acquired at 4 cm^–1^ optical resolution over the spectral range of 930–3050 cm^–1^ and stored at 2 cm^–1^ spacing. Preprocessing of the infrared spectra involved truncation to 950–3000 cm^–1^, and exclusion of the 1800–2800 cm^–1^ range, which contains minimal relevant absorbance in biological media. Each spectrum was represented as an absorbance vector across wavenumbers and L2-normalized unless otherwise specified.

To systematically compare workflow-related effects on IMF, we compared each preanalytical workflow variation against a reference workflow by differences between condition-wise mean IMFs. Specifically, we computed the difference between the mean IR molecular fingerprint of samples processed according to a defined operating procedure and the mean fingerprint of samples subjected to the corresponding preanalytical workflow variation. Shaded gray regions on these graphs indicate wavenumber-resolved between-person biological variability estimated in a previous study,^26^ shown as a reference range for typical IMF heterogeneity in clinical sampling settings collected under SOP.

To further assess whether the sampling parameters affected IMF in a quantitative and multivariate manner, we performed pairwise binary classification (reference versus variation) using binomial logistic regression. Spectral data from the selected regions were first L2-normalized, then Z-score standardized. Principal component analysis (PCA) was fitted on the training data only, and all principal components except the last one were retained as model inputs; the corresponding training-set standardization parameters and PCA loadings were subsequently applied to the test data to prevent information leakage. The classifier was trained using lasso regularization, with a binomial distribution and logit link (Alpha = 1e^–5^, Lambda = 0). Model performance was evaluated using 20 repeats of stratified 5-fold cross-validation. For each fold, predicted probabilities for the positive class were obtained from the test set and stored as out-of-fold predictions. Within each repeat, the out-of-fold predictions from the five test folds were combined to generate one pooled prediction set, from which a single pooled ROC-AUC value was calculated.

To further estimate uncertainty, bootstrap resampling was performed separately on the pooled out-of-fold predictions from each repeat (B = 1000, sampling with replacement). ROC-AUC was recalculated for each bootstrap sample, and resamples containing only one class were excluded. For each repeat, the 95% confidence interval was estimated from the 2.5th and 97.5th percentiles of the bootstrap ROC-AUC distribution. The final reported ROC-AUC and confidence intervals were obtained by averaging the bootstrap mean ROC-AUC values and corresponding confidence bounds across the 20 repeats.

For statistical comparison between preprocessing conditions, pairwise comparisons were performed on the 20 repeat-wise pooled ROC-AUC values using the Mann–Whitney U test. Resulting p-values were adjusted for multiple testing using the Bonferroni correction.

To integrate the results across all investigated preanalytical variables into a single, comparable effect-size scale, workflow-induced spectral deviations were summarized over the full wavenumber range (excluding the silent region). Specifically, for each reference-versus-variation comparison, wavenumber-resolved L2-normalized absolute differences |ΔIMF(ν̃)| were computed and aggregated by the mean absolute difference across wavenumbers (Figure 6). To make this aggregation comparable across spectral regions and across workflow parameters, spectra were Z-score standardized per wavenumber prior to computing the mean absolute differences. This expresses deviations relative to the typical variability and prevents regions with larger variance from dominating the integrated magnitude purely due to scale. The resulting summary values provide a unified basis to compare effect magnitudes across all tested conditions. For reference, between- and within-person biological variability (derived from previously established variability estimates^26^) is shown in comparison to other workflow parameters tested, to contextualize preanalytical effect sizes relative to population-level heterogeneity.

## Results

We systematically examined how different clinical blood sampling and preanalytical processing parameters affect infrared molecular fingerprints of cell-free blood measured by Fourier transform infrared (FTIR) spectroscopy. Across more than 600 individual spectroscopic measurements, we quantified the impact of blood collection tube type, nominal and insufficient tube filling, precentrifugation delays and centrifugation conditions, freezing and thawing procedures including repeated freeze–thaw cycles, and post-thaw delays prior to measurement on infrared (IR) spectral fingerprints (Figure 1A).

**Figure 1:**
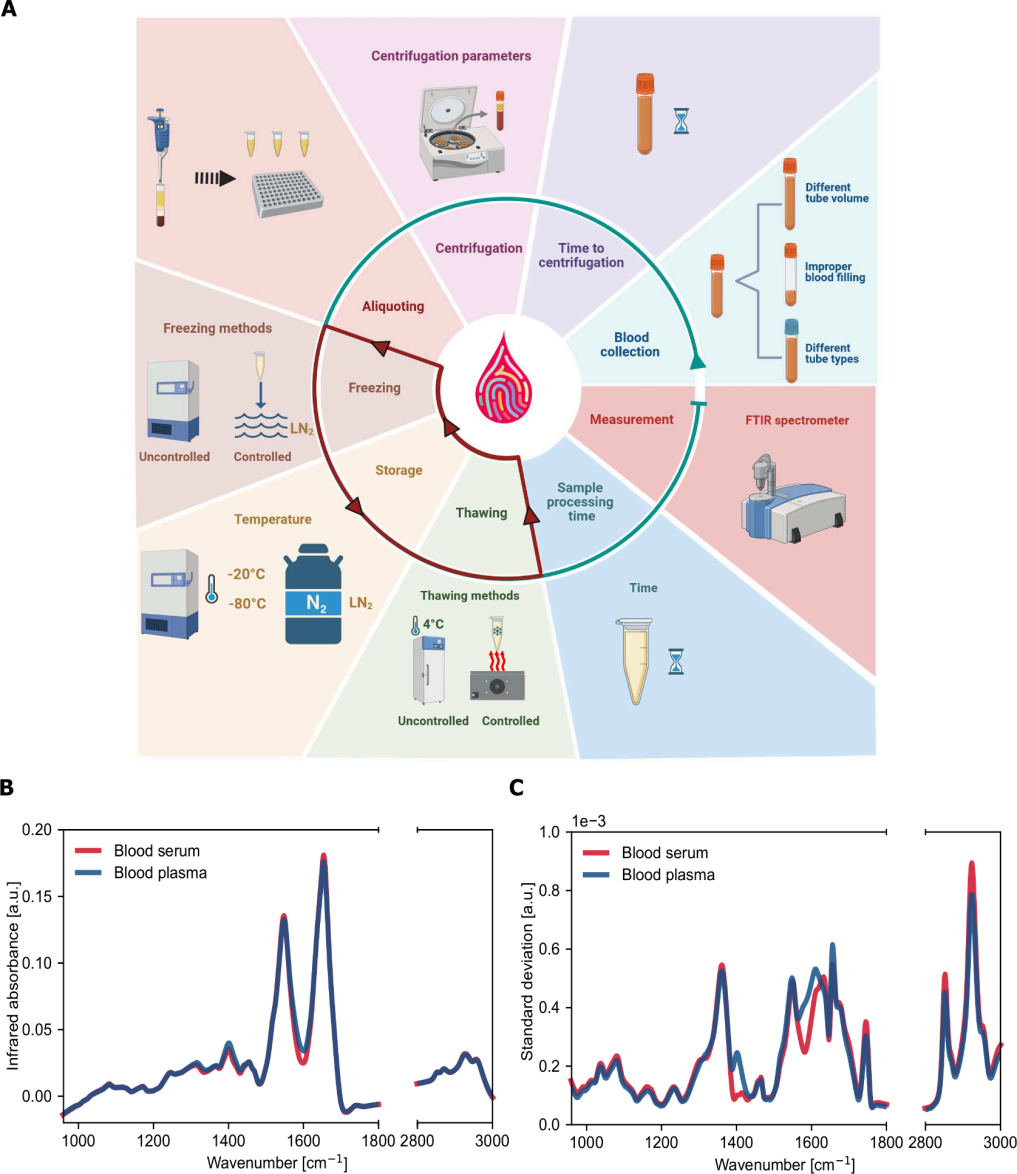
Overview of workflow parameters evaluated for blood-based infrared molecular fingerprinting (IMF). (A) Schematic of the workflow highlighting different clinical and preanalytical variables investigated for their influence on IMF performance. (B,C) Peripheral venous blood from the same individuals was collected in a single blood draw and processed to plasma and serum: (B) L2-normalized infrared molecular fingerprints of two blood-based matrices (EDTA-plasma, serum) of 33 individuals from a previous study^26^ reveal high overall similarities across the measured infrared spectra. (C) Wavenumber-resolved standard deviation with matrix-dependent variability across the spectral range.

Predefined clinical and preanalytical effects were systematically compared across parameters by quantifying deviations from a reference workflow (standard operating procedure, SOP - as defined in Methods). Specifically, we computed the difference between the mean IR molecular fingerprint of samples processed according to the predefined reference SOP and the mean fingerprint of samples subjected to controlled preanalytical workflow variations. To evaluate the impact of each variable and assess the separability introduced by each variable, we performed pairwise binary classification between samples under two different, comparable conditions, using the area under the curve (AUC) summarizing the receiver operating characteristic (ROC) to quantify classification performance. Ultimately, we integrated the results across all variables and benchmarked the amplitude of preanalytical effects against biological variability, both within-individual variation over time and between-individual differences, thus contextualizing the amplitude of controlled, workflow-introduced deviations in clinically realistic laboratory settings and populational studies.

### Differences between Blood Plasma and Blood Serum

To evaluate the suitability of different blood-derived matrices for robust blood-based IMF under clinically relevant conditions, we quantified IR spectral variability in peripheral venous blood drawn from the same individuals during a single venipuncture and processed in parallel to either plasma or serum. L2-normalized infrared molecular fingerprints from EDTA-plasma and serum exhibited characteristic IR spectral signals associated with the major molecular classes in cell-free blood – proteins, carbohydrates, metabolites, lipids, and lipoprotein particles,^14^ with expected similarities between both matrices across the measured spectral range and comparable IR spectral signal amplitudes (Figure 1B).^26^ Moreover, assessment of the IR spectral variability demonstrated that serum and plasma exhibit similar overall spectral variability, with matrix-specific differences indicated by the standard deviation profiles (Figure 1C). These matrix-specific differences primarily arise from EDTA in plasma, which alters spectra near 1400 and 1600 cm^–1^. The spectral similarities suggest that both serum and plasma are, in principle, suitable matrices for reproducible IMF analyses. Plasma was selected for subsequent analyses of preanalytical factors affecting IMF because it is among the most widely used specimens and often preferred matrix in biomedical research.^21^ Several large-scale population omics resources were generated using blood plasma,^15,29,39^ and evidence from serum metabolomics and proteomics suggests that serum is highly susceptible to the coagulation-related alterations.^11,28,31,35,38^

To further characterize the technical and biological sources of spectral variability inherent to IMF, we compared mean infrared molecular fingerprints and their associated standard deviations across a reference sample set comprising the same blood plasma and serum samples, pooled human blood plasma (QC serum), water, and DMSO_2_ (Figure S1). Standard deviation profiles revealed that biological between-person variability is the dominant source of spectral variation in both plasma and serum, substantially exceeding the technical variability captured by repeated QC serum measurements, water, and DMSO_2_ replicates (Figure S1B). Principal component analysis of the non-normalized IMF measurements confirmed that technical variability is small, as shown by the tight clustering of QC serum, water, and DMSO_2_ replicates, while the broad dispersion of plasma and serum samples predominantly reflects biological between-person variability rather than matrix-specific or technical sources of spectral variation (Figure S1C).

### Impact of Blood Collection Tube Type and Fill Volume

Peripheral blood collection is the first step in the clinical preanalytical workflow, and its reproducibility is critical because variability introduced at this stage can propagate through all subsequent handling and analytical steps. In routine clinical practice, venous blood may be collected into a variety of tube types that differ in nominal tube volume (i.e., absolute tube volume) and the amount to which the medical personnel is in practice filling the tube (hereafter ”fill volume”), additives (e.g., EDTA for plasma preparation), and manufacturer. To quantify how these variables influence IMF, we computed differential IR fingerprints from FTIR spectra obtained from samples collected in (i) tubes with different nominal volumes, (ii) partially filled tubes (underfilling), and (iii) different tube types (additives and suppliers) (Figure 2A–C) according to the experimental setting in Table S1. To determine whether these tube-related differences are detectable at the IR fingerprint level, we trained variable-specific binary classifiers for each condition and summarized performance by AUCs (Figure 2D).

**Figure 2:**
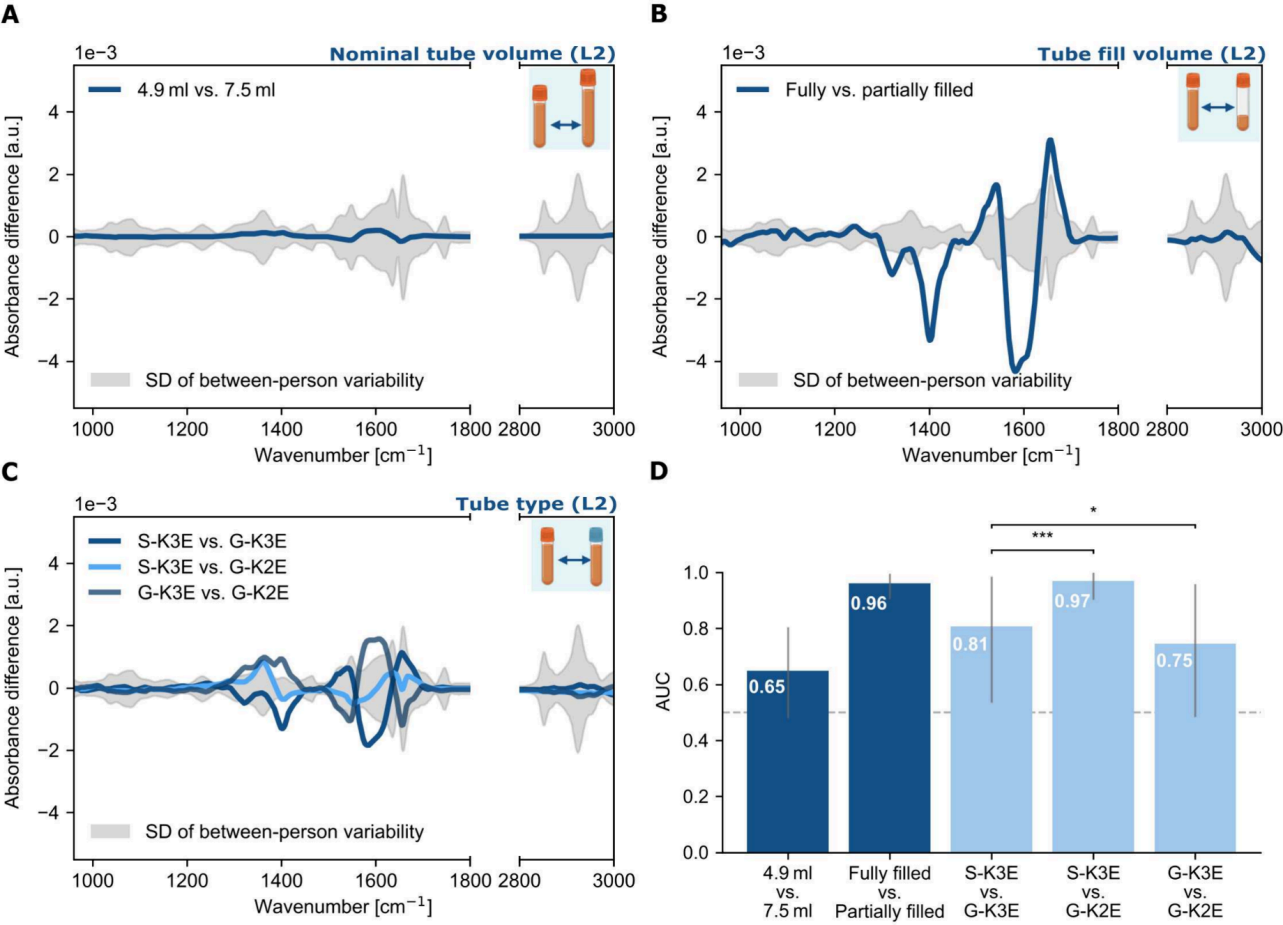
Influence of blood collection tube (nominal) volume, fill volume, and tube type on IMF. (A–C) L2-normalized differential infrared molecular fingerprints (absorbance difference [a.u.]) relative to the SOP reference with the following tube parameters: (A) Effect of nominal tube volume (4.9 mL vs 7.5 mL). (B) Effect of fill volume (fully filled versus partially filled tubes). (C) Effect of EDTA-plasma tube type as pairwise differential fingerprints: S-K3E (Sarstedt K3-EDTA, 1,6 mg/mL), G-K2E (Greiner K2-EDTA, 1,8 mg/mL), and G-K3E (Greiner K3-EDTA, 1,8 mg/mL). Shaded gray areas indicate the standard deviation of the between-person variability. (D) Pairwise classification performance for distinguishing SOP-prepared samples from samples subjected to altered tube-related workflows (mean AUC); dashed gray line marks AUC = 0.5 (chance-level discrimination), and error bars show the 95% confidence interval of the bootstrap AUC distribution. Significance brackets denote pairwise statistical comparisons against the reference condition (ns *p* ≥ 0.05, *p < 0.05, **p < 0.01, ***p < 0.001). Figure S2 in the Supporting Information shows the corresponding spectra for serum, while Figure S3 shows the plasma spectra of the same experimental data visualized without normalization and with min–max-normalization.

We first tested whether different nominal tube volumes, when using the same collection tube type, influence the resulting IR molecular fingerprints. For both S-CAT serum and S-K3E plasma tubes of differing volumes, no significant effect was observed. The L2-normalized (Figure 2A and Figure S2A′), non-normalized (Figure S2A and Figure S3A) and min–max-normalized (Figure S2A″ and Figure S3A′) differential IR fingerprints comparing samples collected in 4.9 mL versus 7.5 mL tubes remained within the standard deviation of the between-person variability, indicating that any capacity-related effect, if present, is smaller than the intrinsic analytical variability. Consistently, binary classifiers trained to distinguish samples collected in 4.9 mL versus 7.5 mL tubes showed limited discriminative performance, suggesting that tube fill volume has a minimal, marginally detectable effect on IMF (AUC 0.65 for S-K3E plasma) (Figure 2D).

Although nominal tube volume had a negligible impact relative to other sources of variability, tube manufacturers specify a minimum acceptable fill volume for plasma and serum tubes. This is analytically relevant because underfilling alters the effective concentration of tube additives. For example, if one study site routinely fills tubes close to the lower limit of the recommended range, whereas another fills them closer to nominal volume, systematic differences in additive concentrations may introduce preanalytical bias in comparisons across study sites and protocols. To quantify this effect, we compared L2-normalized (Figure 2B and Figure S2B′), non-normalized (Figure S2B and S3B) and min–max-normalized (Figure S2B″ and Figure S3B′) differential IR molecular fingerprints between fully filled (4.9 mL/4.9 mL) versus partially filled (2.5 mL/4.9 mL) S-CAT and S-K3E tubes. In contrast to nominal tube volume comparisons, incomplete filling led to pronounced changes in the resulting IR molecular fingerprints of S-K3E plasma. Differential spectra exceeded the standard deviation of the between-person variability across multiple wavenumber regions. Consistently, binary classifications between fully and partially filled tubes reached high discrimination (AUC of 0.96 for S-K3E plasma, Figure 2D). Altogether, these results identify the effect of blood collection tube filling during clinical blood draw as a substantial source of preanalytical variation in IMF of EDTA plasma samples.

As different clinics and laboratories routinely use EDTA tubes from different manufacturers, we next examined whether variations in the anticoagulation additive composition, specifically EDTA concentration and the number of potassium counterions (K2 versus K3), led to measurable alterations in IMF. We compared plasma samples collected in S-K3E tubes (1.6 mg/mL EDTA) with those collected in G-K3E and G-K2E tubes (both 1.8 mg/mL EDTA). Moreover, the tube filling mechanism differs between the two brands (Sarstedt manual aspiration versus Greiner tubes via vacuum). L2-normalized differential IR fingerprints showed tube-specific spectral deviations that exceeded the standard deviation of the between-person variability across multiple wavenumber regions (Figure 2C). The spectra without normalization and with min–max-normalization are shown in Figure S3C–C′. In line with this, binary classification between S-K3E versus G-K3E tubes delivered an AUC of 0.81, and discrimination between S-K3E versus G-K2E tubes yielded an AUC of 0.97. Notably, G-K3E and G-K2E tubes which contain the same EDTA concentration but differ only in counterion composition were distinguishable (AUC 0.75), indicating that EDTA tube chemistry alone can introduce systematic, detectable variation for plasma IMF.

Overall, these findings suggest that whereas certain variations in blood collection (e.g., nominal tube volume) have negligible effects on IMF of serum and plasma, other factors—involving blood collection tube underfilling, anticoagulant type, and anticoagulant concentration—can substantially alter the molecular information encoded in infrared molecular fingerprints.

### Influence of Delays before Centrifugation and Different Centrifugation Regimes

Centrifugation separates cellular components from cell-free serum or plasma. In routine clinical workflows, however, the interval between phlebotomy and centrifugation, as well as the centrifugation settings (relative centrifugal force and duration), vary across laboratories and clinical sites, as immediate processing is not always practical or feasible. Such deviations are analytically relevant because ongoing cellular activity (e.g., metabolism, leakage, and release of intracellular components during delay times) can alter the composition of blood plasma. We therefore systematically quantified the effects of defined precentrifugation delays (Figure 3A–A′) and different centrifugation regimes (Figure 3B–B′) on blood plasma IMF.

**Figure 3:**
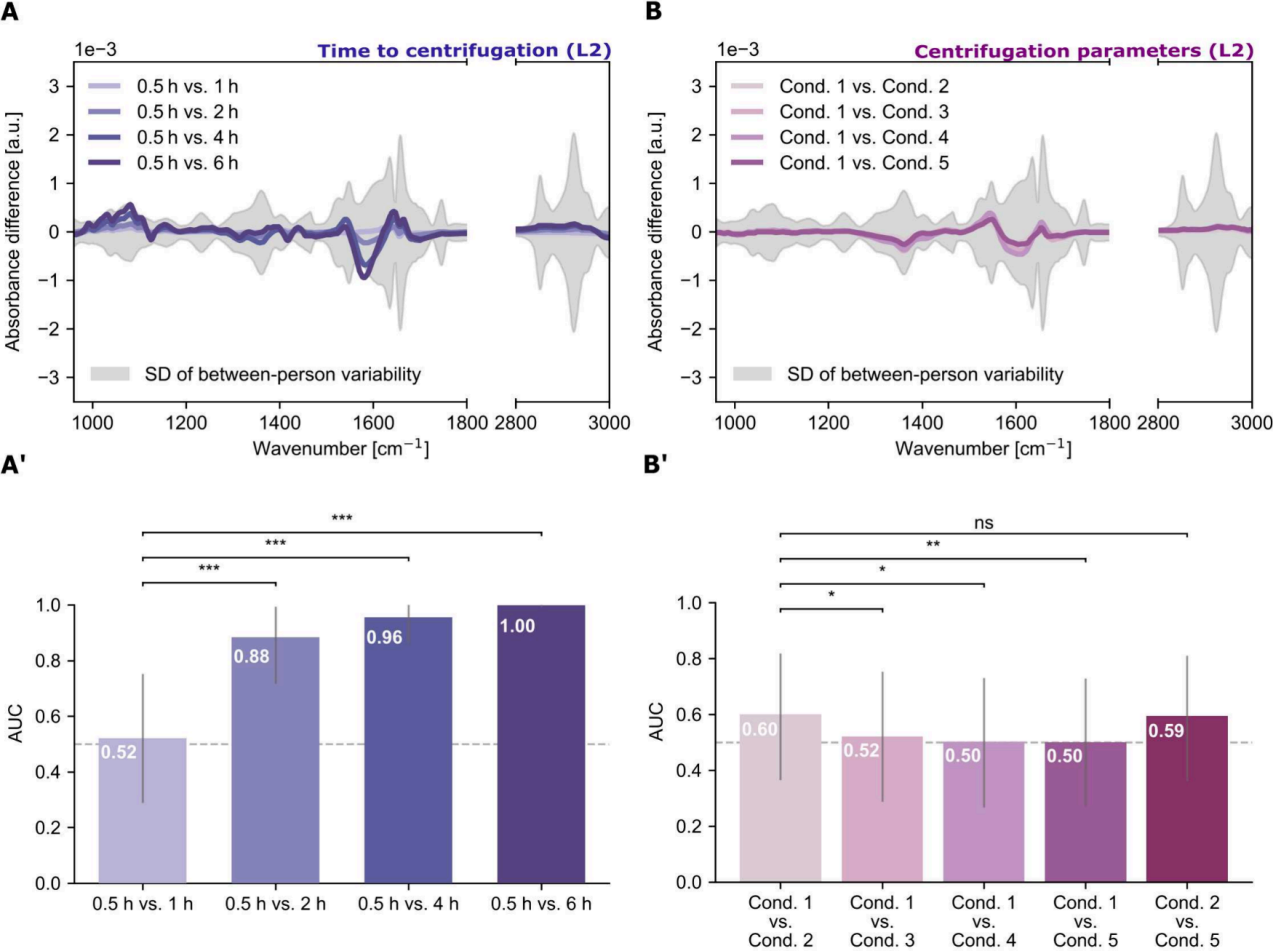
Effects of precentrifugation delays and centrifugation parameters on IMF of EDTA plasma. (A–A′) Impact of precentrifugation delay (time from blood draw to centrifugation), (B–B′) Impact of centrifugation regime. Centrifugation parameters (relative centrifugal force and duration) for condition 1–5 (Cond. 1–5) can be retrieved from Table S2. (A, B) L2-normalized differential infrared molecular fingerprints (absorbance difference [a.u.]) relative to the reference workflow (0.5 h-delay for (A) and 2000 g × 10 min for (B)). Shaded gray areas indicate the standard deviation of the between-person variability. (A′, B′) Pairwise classification performance distinguishing reference-prepared samples from samples subjected to altered precentrifugation delay times or centrifugation parameters (mean AUC). The dashed gray line marks AUC = 0.5 (chance-level discrimination), and error bars represent the 95% confidence interval of the bootstrap AUC distribution. Significance brackets denote pairwise statistical comparisons against the reference condition (ns *p* ≥ 0.05, * *p* < 0.05, ** *p* < 0.01, *** *p* < 0.001). Figure S4 in the Supporting Information shows the corresponding spectra of the same experimental data visualized without normalization and with min–max-normalization.

To quantify the impact of precentrifugation delays of whole-blood, blood collection tubes were kept at room temperature and centrifuged either after 0.5 h (reference) or with up to 6 h delay. Delay-associated changes were assessed using L2-normalized (Figure 3A), non-normalized (Figure S4A), and min–max-normalized (Figure S4A′) differential IR fingerprints relative to the 0.5 h reference values. Spectral deviations systematically increased with delay time. Binary classifiers distinguishing samples with a delay of 0.5 h versus those centrifuged 1 h after collection were not effectively separating the two groups of spectra (AUC 0.52) (Figure 3A′). In contrast, delay times of 2–6 h yielded higher AUCs, reflecting increasingly detectable alterations in plasma molecular fingerprints. Delay times of 2 or 6 h before centrifugation were detected with a high degree of confidence (AUC 0.88–1.00) (Figure 3A′). Notably, delayed centrifugation after blood draw introduced systematic, time-dependent changes in plasma IMF. These effects, however, remained largely within the between-person biological variability but can be detected using machine learning approaches.

We next tested whether variability in venous blood centrifugation settings—specified by manufacturers’ recommendations and differing slightly across brands—affects IMF. Using S-K3E plasma, we tested four centrifugation regimes spanning from 1000 g for 10 min to 3000 g for 15 min, with 2000 g for 10 min as the reference (Cond. 1). Across all regimes, L2-normalized (Figure 3B), non-normalized (Figure S4B), and min–max-normalized (Figure S4B′) differential IR fingerprints remained within the standard deviation of the between-person variability. Consistently, pairwise classifiers performed near chance (AUC slightly above 0.5) (Figure 3B′). Thus, within the tested regimes, centrifugation force and duration had a negligible effect on IMF results.

Taken together, precentrifugation delays exceeding 1 hour, which may easily occur in routine clinical practice, introduced systematic, analytically detectable changes in plasma IMF that increased over delay time, whereas tested variation in the centrifugation settings did not result in analytically significant alterations.

### Freeze–Thaw and Storage Effects on IMF

Blood-based samples used for spectroscopic analyses, especially those from laboratories or biobanks, are typically stored at −80 °C and later on retrieved for retrospective analyses, a workflow that may lead to further aliquoting and thus freeze–thaw cycles. Although the effects of freezing and thawing on molecular sample integrity have been extensively examined in metabolomics and proteomics,^6,8^ their impact on IMF has not been systematically investigated. To address this, we quantified IR fingerprint changes as a function of repeated freeze–thaw cycles (Figure 4A-A′) and further examined whether controlled versus uncontrolled freezing/thawing procedures introduce detectable analytical variability (Figure 4B–B′).

**Figure 4:**
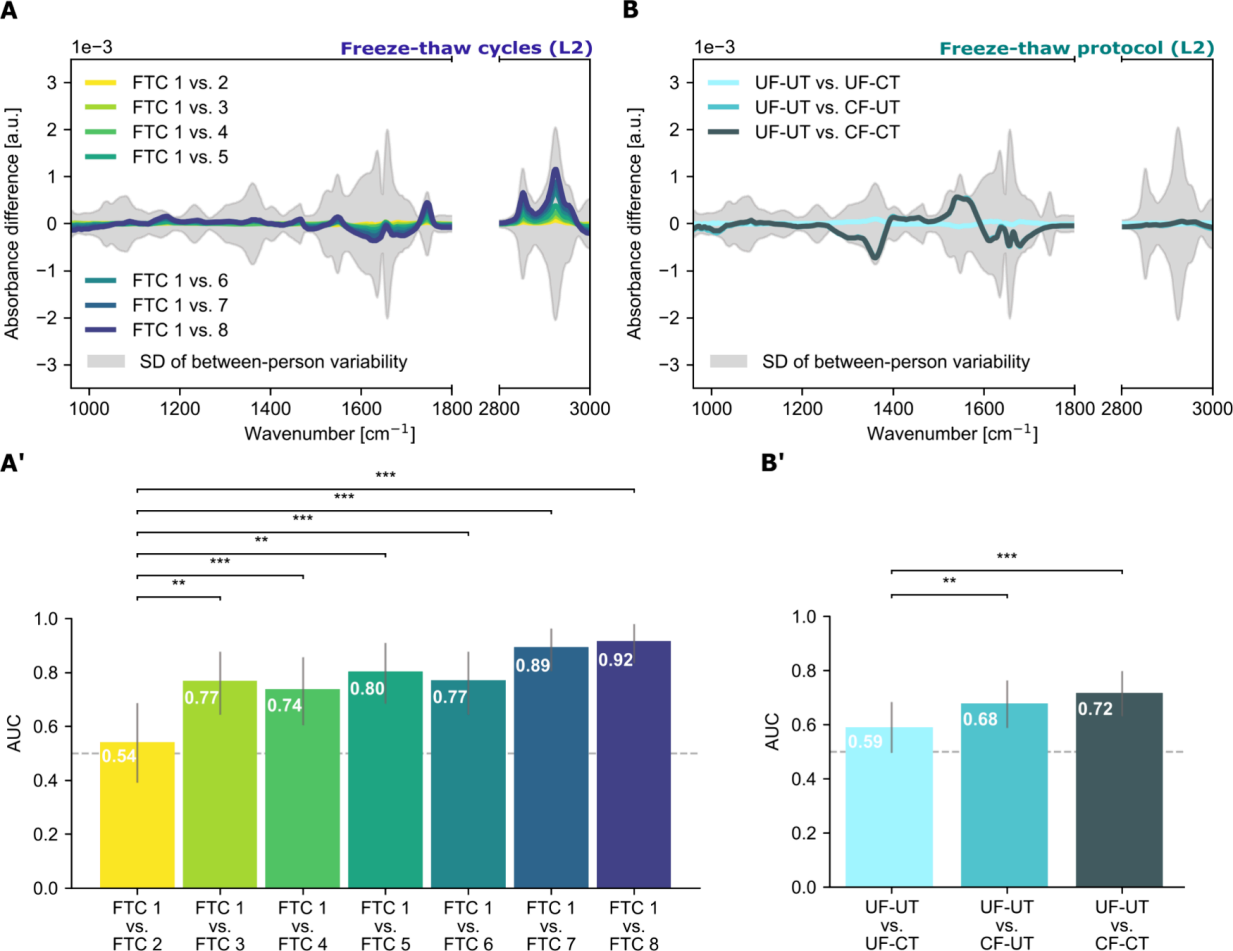
Effects of freeze–thaw cycling and different freezing/thawing procedures on IMF of EDTA plasma. (A–A′) Impact of different numbers of freeze–thaw cycles on IMF. (B–B′) Impact of different freezing and thawing protocols on IMF. (A, B) L2-normalized differential fingerprints (absorbance difference [a.u.]) relative to the reference condition (one freeze–thaw cycle (FTC) for (A) and uncontrolled freezing and thawing (UF-UT) after two FTCs for (B)) comparing reference workflow samples with samples subjected to repeated freeze–thaw cycles or different, controlled freezing/thawing (CF, CT) workflows (after two FTCs). Shaded gray areas indicate the standard deviation of the between-person variability. (A′, B′) Pairwise classification performance distinguishing reference samples from samples subjected to increasing number of freeze–thaw cycles and altered freezing/thawing conditions (mean AUC ± SD). The dashed gray line marks AUC = 0.5 (chance-level discrimination), and error bars represent the 95% confidence interval of the bootstrap AUC distribution. Significance brackets denote pairwise statistical comparisons against the reference condition (ns *p* ≥ 0.05, * *p* < 0.05, ** *p* < 0.01, *** *p* < 0.001). Figure S5 in the Supporting Information shows the corresponding spectra of the same experimental data visualized without normalization and with min–max-normalization.

With an increasing number of freeze–thaw cycles, deviations from the reference condition (one cycle) increased in a dose-dependent manner in both L2-normalized (Figure 4A), non-normalized (Figure S5A), and min–max-normalized (Figure S5A′) spectra. Repeated freeze–thaw cycling led to cycle-dependent changes across several spectral regions. These systematic spectral changes were further reflected in the performance of binary classifiers trained to distinguish samples subjected to different numbers of freeze–thaw cycles (Figure 4A′). Discrimination between two-cycle samples and the one-cycle reference was weak (AUC 0.54), increased for three to six freeze–thaw cycles (AUC 0.77–0.80), and seven to eight cycles resulted in high separability (AUC 0.89–0.92). Thus, repeated freeze–thawing introduces progressively stronger alterations in IR molecular fingerprints detectable using machine learning classifiers, yet these alterations remain within the standard deviation of the between-person variability.

Beyond the freeze–thaw cycle number, freezing and thawing workflows can vary across laboratories, potentially introducing handling heterogeneity (e.g., through position-dependent temperature gradients when using multiwell racks during passive freezing or thawing). Controlled freezing/thawing devices can minimize such effects, but require specialized equipment not universally available. We compared uncontrolled freezing and thawing (UF–UT) with controlled freezing (CF), controlled thawing (CT), and the combined fully controlled workflow (CF–CT) to determine their influence on downstream IMF analysis. Across all conditions, L2-normalized (Figure 4B) non-normalized (Figure S5B), and min–max-normalized (Figure S5B′) differential IR fingerprints remained within the standard deviation of the between-person variability with binary classification revealing modest but detectable effects: UF–CT and CF–UT were distinguishable from UF–UT with AUCs of 0.59 and 0.68, respectively, and CF–CT could be distinguished from UF–UT with an AUC of 0.72.

Moreover, we systematically investigated the effects of storage temperature and duration on FTIR spectra of pooled human blood serum, particularly relevant for settings lacking – 80 °C infrastructure, given that existing evidence from plasma metabolomics and proteomics^5,22^ suggests that −20 °C storage can introduce significant molecular alterations relative to – 80 °C. To investigate storage effects, over 90 QC serum samples were aliquoted and stored under four conditions: overnight at – 80 °C, at – 20 °C, 4 °C, and room temperature (RT) for durations ranging from 1 to 8 weeks, after which FTIR spectra were compared against the −80 °C reference using Principal Component Analysis (Figure S6 and Table S5). Progressively larger spectral shifts were observed at −20 °C, 4 °C, and for samples stored at room temperature. Specifically, deviation from the −80 °C reference condition increased with storage duration at −20 °C, while samples stored at 4 °C and room temperature showed a clear shift already from week 1 onward, with room-temperature storage causing the largest overall variation. These findings highlight postcentrifugation storage temperature and duration as critical parameters for IMF, underscoring the importance of controlled −80 °C biobanking conditions to minimize storage-related molecular changes.

Overall, controlled freezing/thawing and repeated freeze–thaw cycles introduced spectral changes that remained within the range of between-person variability, yet these effects were still detectable by machine-learning classifiers. This level of sensitivity is particularly relevant for analyses of archived blood-based samples, where freeze–thaw handling is either heterogeneous or incompletely documented.

### Effect of Delays between Sample Thaw and Spectroscopic Measurement

IR molecular fingerprint measurement represents the final step in the experimental workflow. Because FTIR measurements are performed sequentially, thawed samples inevitably undergo different post-thaw standing times prior to spectroscopic measurement. In our setting, sample injection and measurement are automated, whereas sample thaw is performed manually in batches, and measurements are performed consecutively. This leads to inevitable differences in the sample’s standing time, depending on their position within the rack, prior to measurement. To avoid potential measurement drifts, we always randomize the sample run order in all clinical studies, as well as measurements across this study. To evaluate such possible systematic changes, here we specifically quantified the effect of the interval between thawing and FTIR measurement (i.e., premeasurement delay) on IMF using commercially available, pooled human blood serum (Figure 5). The 0–0.5 h time window after thawing served as the reference for calculating L2-normalized (Figure 5A), non-normalized (Figure S7A), and min–max-normalized (Figure S7A’) differential IR fingerprints. Across the 0.5–1 h and 1–2 h time windows, mean differential IR spectra did not exceed the standard deviation of the between-person variability. Samples measured after 2–3 h post-thawing showed spectral deviations that, in the wavenumber range 1200–1400 cm^–1^ and at 1542 cm^–1^, slightly exceeded biological variability. These spectral changes were reflected in classification performance (Figure 5B) that yielded significantly different spectral data already when comparing the 0–0.5 h time window with 0.5–1 h samples, and separability increased with longer premeasurement standing times (AUCs up to 1.0). To interpret these high AUC values, it is important to note that for this experiment, only QC serum without biological variability was used. Hence, the classifier can pick even slight spectral changes with high performance. Altogether, these results show that even relatively short post-thaw standing times prior to FTIR measurement can introduce systematic, analytically detectable alterations in IR molecular fingerprints. The practical impact of this effect may be relevant, yet dependent on the study context and design, and further highlights the importance of randomizing sample run order across measurement batches.

**Figure 5:**
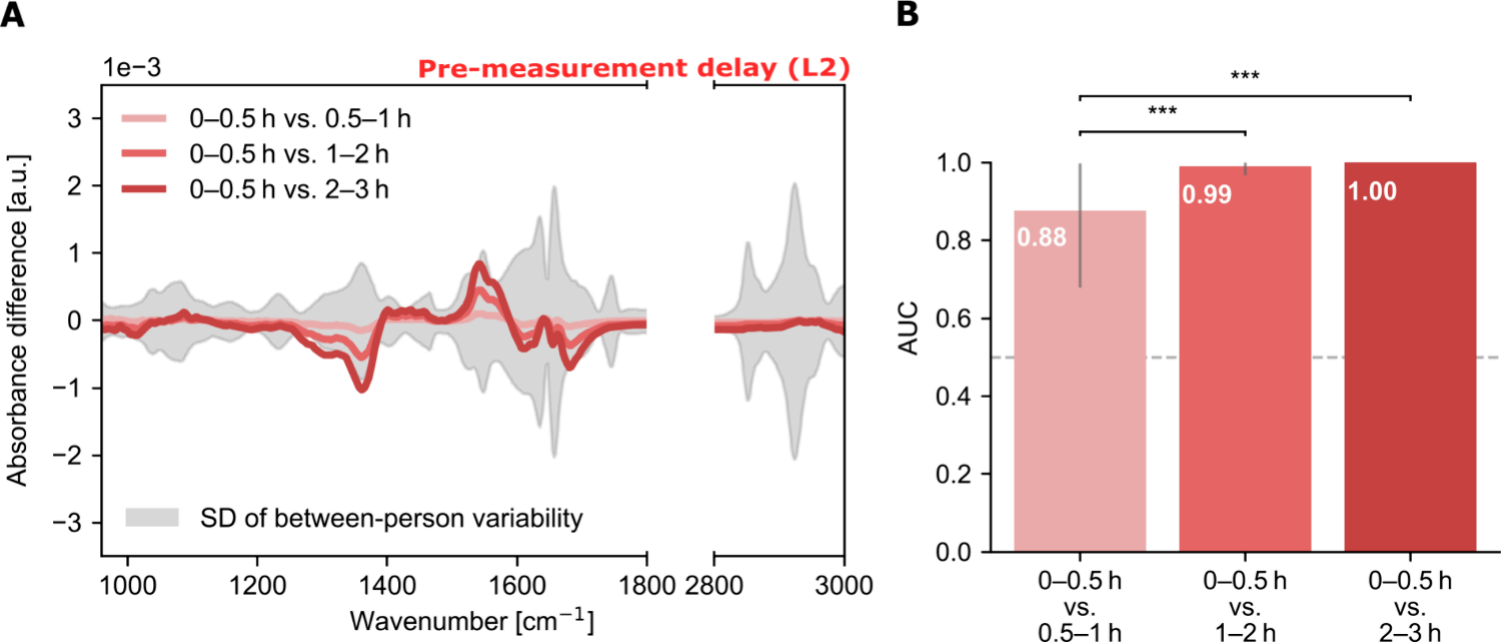
Effects of premeasurement delay time estimated through IMF of pooled blood serum (QC serum). (A) L2-normalized differential fingerprints (absorbance difference [a.u.]) between the mean of reference samples (0–0.5 h premeasurement delay) and those subjected to prolonged premeasurement delays. Shaded gray areas indicate the standard deviation of the between-person variability. (B) Performance of each pairwise classification (mean AUC ± SD) for distinguishing reference samples from samples subjected to longer delay times before measurement. The dashed gray line marks AUC = 0.5 (chance-level discrimination), and error bars represent the 95% confidence interval of the bootstrap AUC distribution. Significance brackets denote pairwise statistical comparisons against the reference condition (ns *p* ≥ 0.05, * *p* < 0.05, ** *p* < 0.01, *** *p* < 0.001). Figure S7 in the Supporting Information shows the corresponding spectra of the same experimental data visualized without normalization and with min–max-normalization.

### Comparing Clinical, Preanalytical and Biological Sources of Variability

The above results demonstrate that clinical and preanalytical handling can measurably alter IMF. Because clinical IMF studies commonly rely on machine learning to compare groups of samples, cohorts, and/or track the same individuals over time, it is critical to assess whether workflow-related spectral changes are within the range of biological variability or exceed it, potentially biasing population-based analyses. Building on our earlier quantification of biological variability in blood-based IR molecular fingerprints,^26^ we benchmarked the magnitude of each examined preanalytical effect against biological variability - within-person variability over time and between-person differences at a single cross-sectional time point. Wavenumber-resolved L2-normalized absolute differences for all tested workflows and biological variability were examined to quantify how each parameter’s contribution to spectral variability across the measured FTIR spectra (Figure 6A). For comparison across parameters, these wavenumber-resolved differences were further condensed into a single metric - mean absorbance difference Z-scores across the spectral range (Figure 6B).

**Figure 6:**
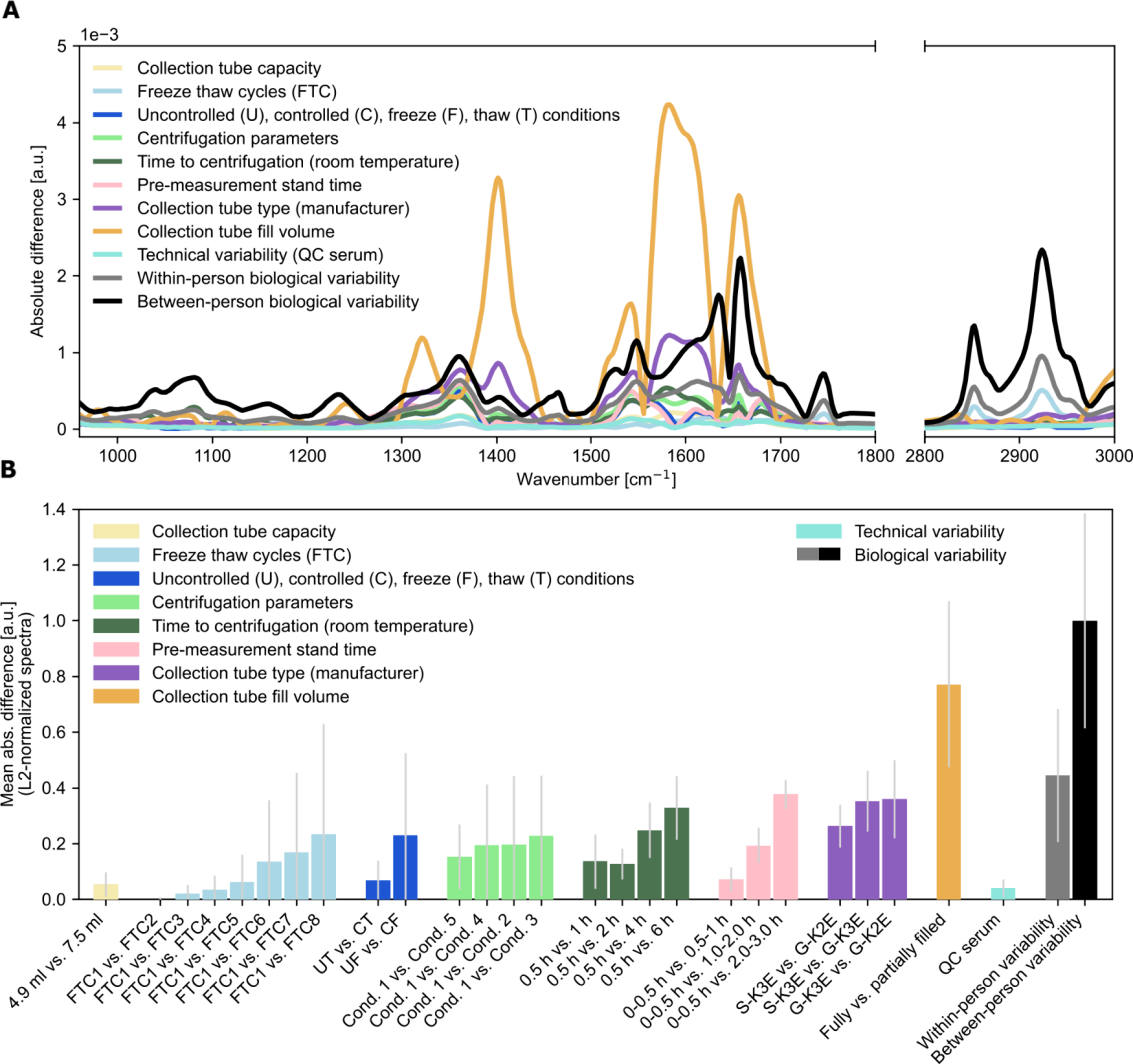
Comparison of clinical and preanalytical parameters relative to biological variability in IMF. (A) Wavenumber-resolved L2-normalized absolute absorbance differences [a.u. ± SD] associated with defined clinical and preanalytical workflow differences, as well as with within-person and between-person biological variability. (B) Z-score standardized mean absorbance differences across the spectral range [a.u.] for each parameter tested, along with biological variability. The results are derived from the wavenumber-resolved L2-normalized absolute FTIR spectral differences.

Between-person variability showed the largest mean absorbance differences (Z-score = 1), and within-person variability followed the similar overall variability spectral pattern but with a reduced amplitude. Among the preanalytical factors tested, incomplete tube filling resulted in the largest mean absorbance differences, followed by tube type, with G-K3E tubes introducing the biggest difference. A 6 h precentrifugation delay resulted in differences approaching within-person biological variability, whereas delay times of 4 h or less remained below this benchmark. In contrast, variations in centrifugation regimes did not exceed within-person variability. Freeze–thaw handling introduced differences also smaller than biological variability. The overall impact of differing freezing and thawing procedures also led to differences below biological variability, and although the deviations systematically increased with the number of freeze–thaw cycles, the variability still remained below biological variability even after eight freeze–thaw cycles. Nominal blood collection tube capacity led to lowest overall IR spectral variability among tested conditions.

Overall, between-person biological variability was the largest source of IMF variation and exceeded the effects of the preanalytical factors tested. The main exception was incomplete blood collection tube filling, which introduced spectral deviations approaching the magnitude of between-person variability.

## Discussion

Infrared molecular fingerprinting (IMF) of blood-derived matrices offers an analytical readout with value for clinical *in vitro* diagnostics. However, its translational utility depends on controlling preanalytical variability that can introduce systematic bias in downstream data analyses. Here, we quantified how clinically relevant handling parameters across sample collection, processing, storage, and measurement timing affect FTIR-derived results. As IMF is ever more widely used in clinical context, we benchmarked sample handling-induced spectral changes versus biological variability.^26^

Among the blood-collection-related factors, we identified the most prominent IMF deviation originating from improper tube filling during the blood draw. Proper filling of blood sampling tubes is highly relevant because underfilling directly changes the concentrations of clot activators or anticoagulants (e.g., silicate and gel or EDTA) in the sample, thereby potentially introducing systematic spectral differences. Insufficient fill volume is also known as a preanalytical error arising in the rush of clinical routine. Although anticipated, this effect has not been previously quantified for IMF and thus has not been coherently implemented in IR profiling. From a practical perspective, complete filling of blood draw tubes is a parameter that is fairly easily implementable in phlebotomy clinical routine workflows, and targeted training of medical personnel to ensure correct fill volume may effectively contribute to sample quality and not only to IMF robustness but possibly also ensure robust outcomes of other clinical chemistry parameters. Importantly, incomplete tube filling led to changes comparable to the amplitude of between-person variability, indicating its direct relevance and possible impact on biologically relevant changes if it is not tightly controlled.

We also observed differences between EDTA plasma tubes (K2- versus K3-EDTA and across suppliers), showing that “EDTA plasma” is not equivalent unless the tube type, manufacturer, and fill volume are standardized. This is also critical for comparisons across clinical sites, studies, and biorepositories, where tube-related effects can mask biological differences. In prospective sample collections, such bias can be realistically avoided with harmonized collection consumables.

In contrast, differences in centrifugation parameters had only a minor impact on molecular fingerprint variability, whereas delaying centrifugation from the time of blood draw progressively increased spectral deviations. This is consistent with previous studies^23^ and with the fact that precentrifugation standing allows for ongoing enzymatic reactions and cellular activity prior to separation from cells. To reduce these effects, the delay time between blood draw and centrifugation should be minimized and documented, as recommended for other analytical platforms.^19^ Different centrifugation parameters may affect the number of platelets remaining in plasma. However, our FTIR workflow (integrating particle filters) is rather insensitive to platelet counts, as previously shown in a large scale populational study.^13^ Therefore, we recommend limiting standing time to less than 1 h, a time window that should be feasible within clinical workflows. Refrigerated storage of blood tubes prior to centrifugation might further increase sample quality, however, this is not applicable for serum. When compared to a clinical setting with biological variability as a benchmark, changes introduced by delays up to 4 h remained below within-person variability, whereas those introduced by delays up to 6 h approached it.

The results of the effects of freezing and thawing, unavoidable steps in most clinical research and biobank workflows, demonstrated that controlled versus uncontrolled freezing and thawing introduced only modest differences in IMF compared to the intrinsic measurement variability. In contrast, series of repeated freeze–thaw cycles led to systematic spectral drifts that increased with the number of cycles. Prior metabolomics and proteomics work similarly reported that repeated freeze–thaw cycles can introduce systematic molecular changes, particularly when subtle changes are targeted.^20,36^ Importantly, even though the magnitude of freeze–thaw-induced spectral variation remained smaller than interindividual biological variability in our data set, such effects can still bias and confound studies targeting relatively subtle biological differences (e.g., early disease changes, or longitudinal within-person comparisons). Therefore, the samples should be stored at – 80 °C under controlled biobanking conditions and the number of freeze–thaw cycles should be minimized by aliquoting and documenting each sample aliquot. Comparisons between cohorts that differ in freeze–thaw cycle history should therefore be avoided, or freeze–thaw count should be explicitly matched. Notably, post-thaw standing time of samples prior to FTIR measurement emerged as a relevant source of spectral changes, indicating the impact of workflows and necessitating randomized sample run order. This could be overcome by thawing samples one-by-one immediately prior to measurement. However, such a workflow would require automatization and is not broadly applied in practice.

## Conclusions

The results of these examinations led us to propose the following operational recommendations for performing robust IMF: (i) standardizing tube type, manufacturer, and fill volume; (ii) minimizing time right after blood draw and prior to centrifugation (targeting less than 1 h); (iii) aliquoting samples into rather small volume fractions to avoid repeated freeze–thaw cycles; (iv) comparing only samples with matched numbers of freeze–thaw cycles; (v) standardizing premeasurement time as samples are being prepared for actual measurements and randomizing their order. Furthermore, the amplitude of biological variability is relevant for prioritizing the impact of these steps, with tube filling and tube specification being of the highest priority, followed by timing to centrifugation and post-thaw measurement timing.

Integrating data from over 600 individual measurements, this work is still limited by the number of analyzed samples and by the lack of orthogonal molecular measurements to directly link observed spectral differences to specific molecules. Importantly, this does not undermine the primary analytical utility of IMF, which is to capture reproducible biochemical fingerprints of samples. In infrared spectroscopy, spectral band overlap and correlated signal contributions from proteins, lipids, carbohydrates, and metabolites limit molecule-specific interpretation but still enable sensitive detection of compositional differences between samples.^14^ In line with previous studies in the field of proteomics, metabolomics, or IR spectroscopy,^4,10,24,32^ IMF was resilient to differences in centrifugation protocols but sensitive to a few parameters (e.g., tube effects, precentrifugation and premeasurement delay), providing a practical basis for harmonized, standardized protocols and reduced preanalytical confounding in translational studies. Nevertheless, we have not evaluated the extent to which the studied parameters would be limited to blood-based IMF from certain health deviations, as we did not assess their possible impact on sampling diseased individuals. Collectively, our proposed measures address the dominant sources of handling-induced IMF variation and improve and inform comparability across measurements, sample collections, and clinical studies. Importantly, the measures we identify here align with previously proposed standard operating guidelines for broader clinical blood serum and plasma management.^37^ Reassuringly, biological variability exceeded the variability of most parameters and handling effects examined here, supporting the feasibility of applications in real-world workflows provided that they are controlled for the main variables.

Additionally, the value of the above recommendations—as a guideline for future translational research in clinical studies and IMF—lies in the fact that the conclusions we derived may be generalizable also to other analytical profiling technologies, such as mass spectrometry^18,27^ and NMR,^29,34^ and other liquid biopsy analytics.^2^ It has been reported that such high-resolution technologies often record *ex vivo* parameters as biological artifacts that cannot be opposed by larger sample sizes or statistical adjustments.^30^ Given the high-throughput, low-cost, and ease of operation of FTIR spectroscopy, the approach may also be of relevance for large-scale biorepositories and biobanks to examine the impact of sample quality and possible long-term storage and degradation effects.^5,22^ Benchmarking against biological variability also provides a practical reference for possible biorepository ”quality control” workflows, relating and defining handling- and workflow-related changes to populational variability.

## Supplementary Material



## Notes

Additional details on the experimental design, including listing of the tested workflow variations and measurement counts; an overview of between-person and technical variability in IMF; results from the temperature-dependent storage experiments; absorbance differences of blood plasma samples due to variations in preanalytical parameters, derived from differential molecular fingerprints without L2-normalization and with min-max-normalization, and evaluation of nominal collection tube and fill volume effects on blood serum samples, analyzed with L2- and min-max-normalization and without normalization (PDF)

## §

Transport at Nanoscale Interfaces Laboratory, Empa–Swiss Federal Laboratories for Materials Science and Technology, CH-8600 Dübendorf, Switzerland
