# Supplementary material for: Preanalytical Workflow Establishment for Reproducible Clinical Blood-Based Infrared Molecular Fingerprinting
Source: Anal Chem. 2026 Jun 17;98(25):18722–34. doi: 10.1021/acs.analchem.6c01358 (PMC13325443; doi:10.1021/acs.analchem.6c01358)
Supplement: Supplementary file 1 [file ac6c01358_si_001.pdf]

Preanalytical Workflow Establishment for Reproducible Clinical Blood-Based Infrared Molecular Fingerprinting

Katharina E. Dietmann[1,2,4,6], Guanting Guo[1,6,8], Jacqueline Aschauer[1,3], Tarek Eissa[1,2], Frank Fleischmann[1,7], and Mihaela Žigman[1,2,5,7,*]

[1]Ludwig-Maximilians-Universität München, Department of Experimental Physics - Laser Physics, 85748 Garching, Germany
[2]Max Planck Institute of Quantum Optics, Laboratory for Attosecond Physics, 85748 Garching, Germany
[3]Ludwig-Maximilians-Universität München, University Hospital, 81377 München, Germany
[4]Technical University of Munich (TUM), School of Life Sciences, 85354 Freising, Germany
[5]Ludwig-Maximilians-Universität München, Faculty of Medicine, Institute of Pathology, 80337 München, Germany
[6]These authors contributed equally to this work.
[7]These authors share equal responsibility for the research.
[8]Current address: Empa Transport at Nanoscale Interfaces Laboratory, Empa - Swiss Federal Laboratories for Materials Science and Technology, CH-8600 Dübendorf, Switzerland.
[*]Correspondence: mihaela.zigman@mpq.mpg.de

Supporting Information

Additional details on the experimental design, including listing of the tested workflow variations and measurement counts, are provided in Tables S1-S4 (PDF).

An overview of between-person and technical variability in IMF is shown in Fig. S1 (PDF).

Results from the temperature-dependent storage experiments are shown in Tab. S5 and Fig. S6 (PDF).

Absorbance differences of blood plasma samples due to variations in preanalytical parameters, derived from differential molecular fingerprints without L2-normalization and with min-max-normalization, and evaluation of nominal collection tube and fill volume effects on blood serum samples, analyzed with L2- and min-max-normalization and without normalization, are outlined in Figures S2-S5 and S7 (PDF).

Supporting Information

Table S1: Experimental design for testing the effects of tube types and sample fill volume.

Nominal tube volume [ml]	Sample volume [ml]	Supplier[a]	Additive[b]	No. of measurements
4.9	4.9	S	gel, CA	33
4.9	2.5	S	gel, CA	17
7.5	7.5	S	gel, CA	17
4.9	4.9	S	K3-EDTA	34
4.9	2.5	S	K3-EDTA	16
7.5	7.5	S	K3-EDTA	16
4.0	4.0	G	K3-EDTA	8
4.0	4.0	G	K2-EDTA	8
10.0	10.0	G	K2-EDTA	8

a: S = Sarstedt; G = Greiner Bio-One
b: CA = clot activator

Table S2: Experimental design for testing the effects of time to centrifugation and centrifugation conditions.

Condition	Relative centrifugal force [g]	Centrifugation duration [min]	Time to centrifugation [h]	No. of measurements
1	2000	10	0.5	13
1	2000	10	1	13
1	2000	10	2	13
1	2000	10	4	13
1	2000	10	6	13
1	2000	10	1–2	13
2	1000	10	1–2	13
3	2000	15	1–2	13
4	3000	10	1–2	13
5	3000	15	1–2	13

Table S3: Experimental design for testing the effects of the number of freeze-thaw cycles and protocols.

Freeze-thaw cycles	Freezing[a]	Thawing[b]	No. of measurements
1	UF	UT	30
2	UF	UT	31
3	UF	UT	31
4	UF	UT	30
5	UF	UT	33
6	UF	UT	29
7	UF	UT	30
8	UF	UT	31
2	UF	UT	9
2	CF	UT	9
2	UF	CT	9
2	CF	CT	9

a: UF/CF = uncontrolled/controlled freezing
b: UT/CT = uncontrolled/controlled thawing

Table S4: Experimental design for testing the effects of premeasurement delays.

Premeasurement delay [h]	No. of measurements
0–0.5	13[a]
0.5–1	9
1–2	18
2–3	20

a: The last three samples of the 60-sample sequence were thawed and placed into the device directly before measurement (to differentiate premeasurement delay from technical drift of the instrument).

Table S5: Effect of storage conditions on IR spectra of pooled blood serum assessed via Mann-Whitney U test: PCA distance to reference storage conditions (–80 °C) with Bonferroni-corrected p-values; significance levels: $^{***}p \leq 0.001$, $^{**}p \leq 0.01$, $^{*}p \leq 0.05$, ns $p > 0.05$. Abbreviations: RT = Room temperature, ON = overnight, adj = adjusted.

Storage temperature	Storage time (weeks)	p	p_{adj}	**Significance**
−20 °C	1	2.72×10^{-3}	3.53×10^{-2}	*
−20 °C	2	4.19×10^{-5}	5.45×10^{-4}	***
−20 °C	4	2.21×10^{-6}	2.87×10^{-5}	***
−20 °C	8	1.10×10^{-7}	1.43×10^{-6}	***
4 °C	1	2.21×10^{-6}	2.87×10^{-5}	***
4 °C	2	2.21×10^{-6}	2.87×10^{-5}	***
4 °C	4	2.21×10^{-6}	2.87×10^{-5}	***
4 °C	8	1.10×10^{-7}	1.43×10^{-6}	***
RT	1	2.21×10^{-6}	2.87×10^{-5}	***
RT	2	2.21×10^{-6}	2.87×10^{-5}	***
RT	4	2.21×10^{-6}	2.87×10^{-5}	***
RT	8	1.10×10^{-7}	1.43×10^{-6}	***
ON storage (−80 °C)	0	6.89×10^{-2}	8.96×10^{-1}	ns

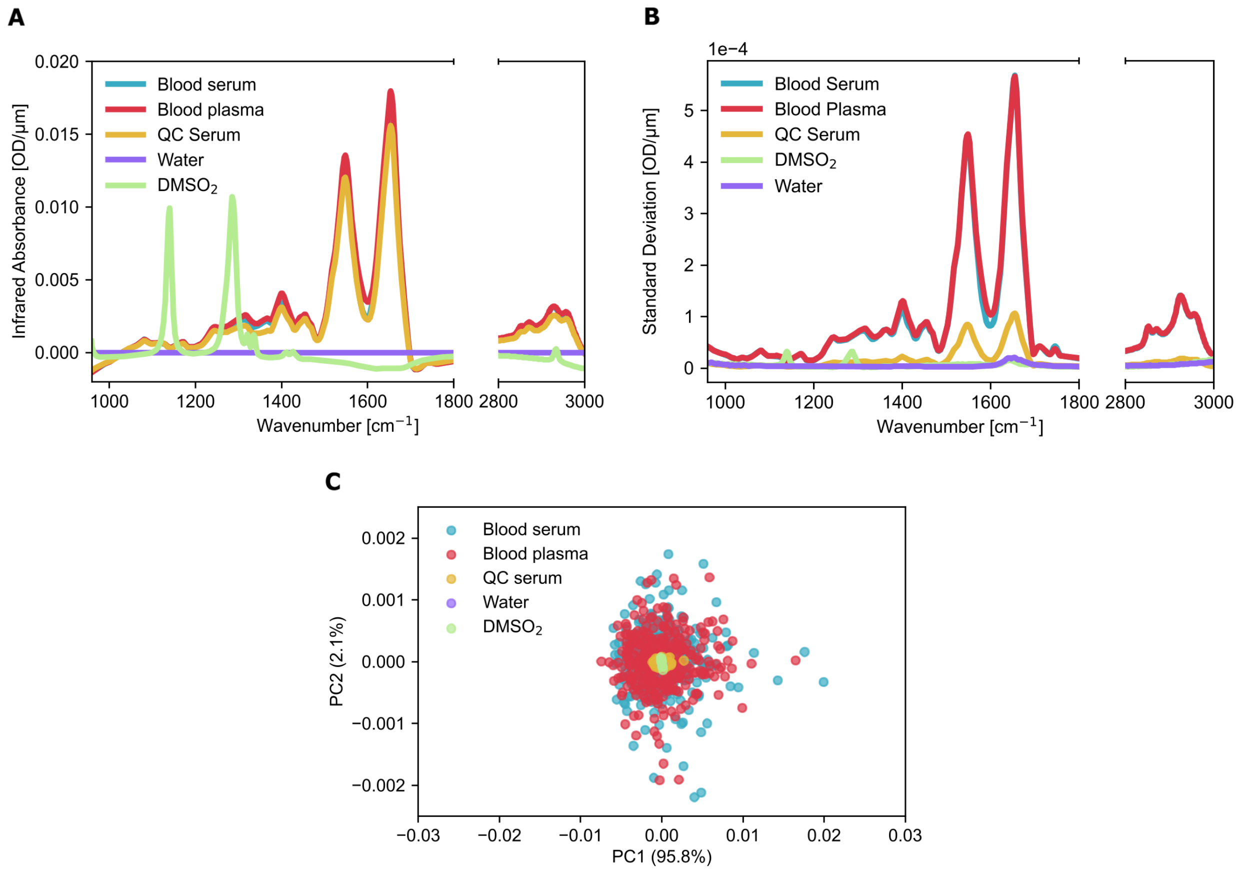

Figure S1: Overview of between-person and technical variability in IMF (non-normalized data). **(A)** Mean infrared molecular fingerprints [OD/µm] of blood plasma and serum samples from 33 individuals from a previous study ([1]), pooled human blood serum (Quality Control (QC) serum), and repeated measurements of $DMSO_2$ (scaled to 20 mg/ml) and water replicates. **(B)** Standard deviation [OD/µm] of the measurements shown in (A), reflecting the biological and technical variability inherent to IMF. **(C)** Principal component analysis (PCA) of the IMF measurements of the same sample set, illustrating the separation of sample types relative to within-group variability.

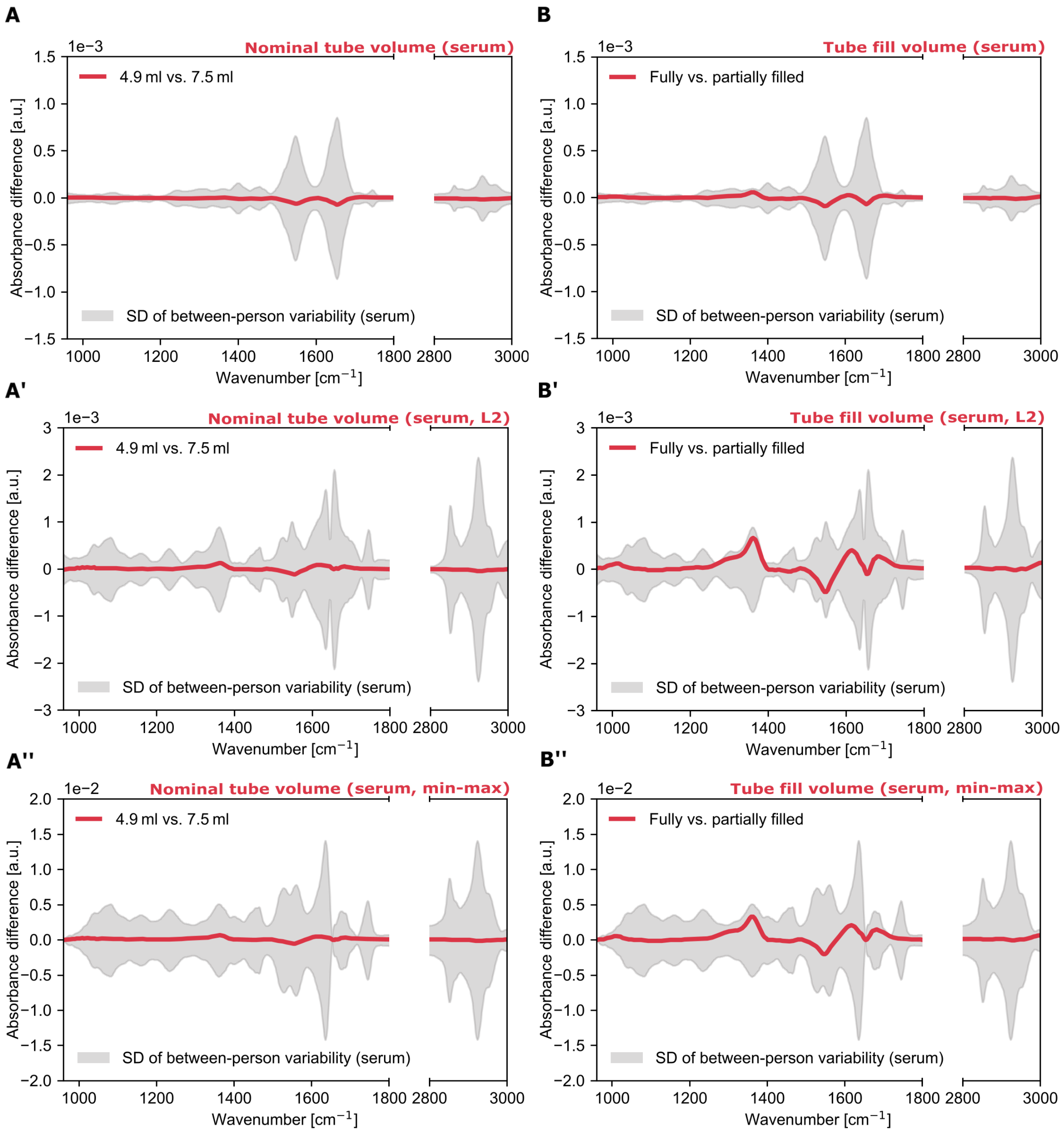

Figure S2: Influence of serum tube (nominal) volume and fill volume on IMF depicted as non-normalized (A,B), L2-normalized (A',B'), and min-max-normalized (A",B") differential infrared molecular fingerprints (absorbance difference [a.u.]) relative to the SOP reference with the following tube parameters: **(A-A")** Effect of nominal tube volume (4.9 ml vs. 7.5 ml). **(B-B")** Effect of fill volume (fully filled versus partially filled tubes). The shaded gray areas indicate the standard deviation of between-person variability.

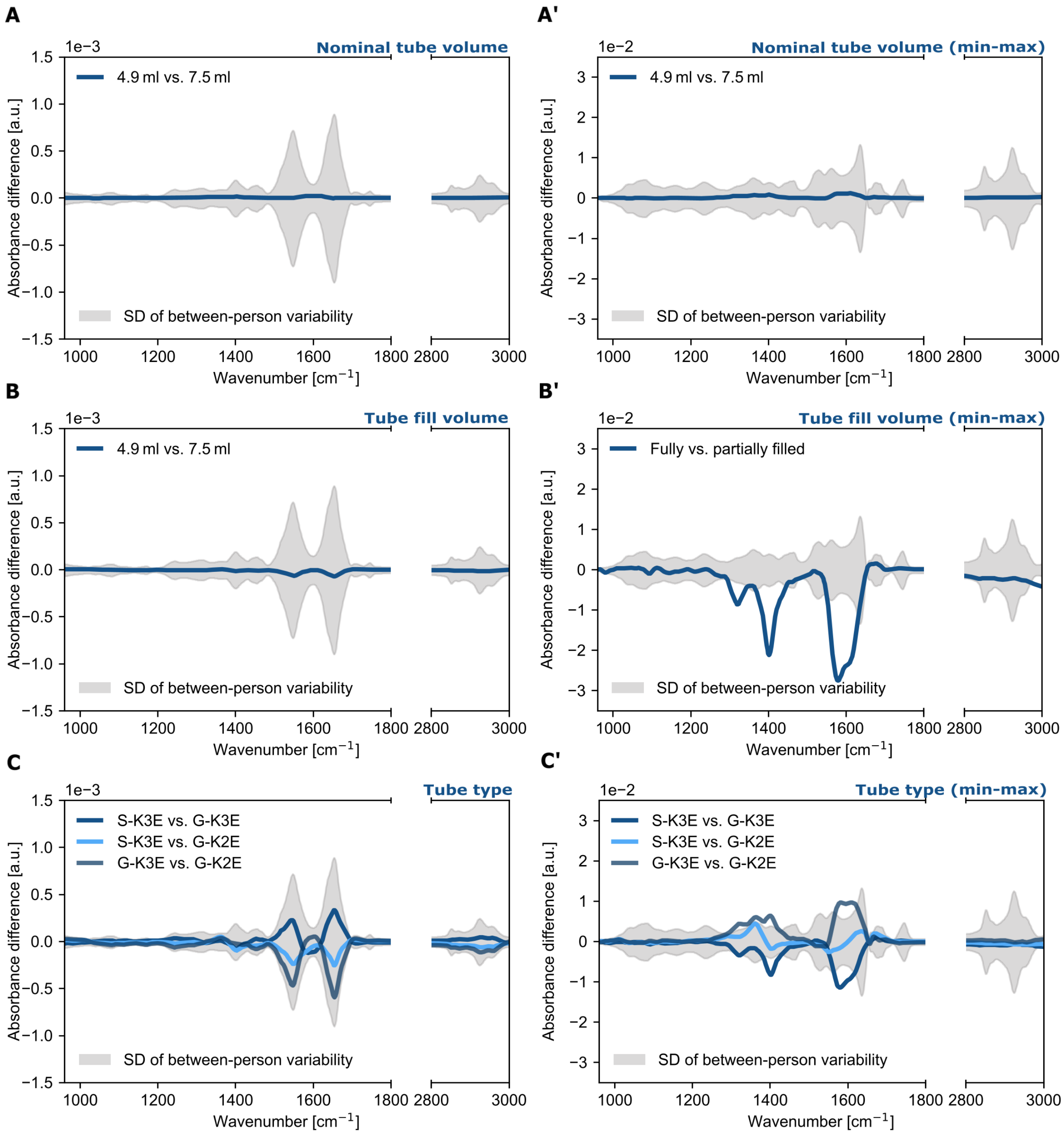

Figure S3: Influence of blood collection tube (nominal) volume, fill volume and tube type on IMF. **(A–C)** Differential infrared molecular fingerprints (absorbance difference [a.u.]) relative to the SOP reference with following tube parameters: **(A-A')** Effect of nominal tube volume (4.9 ml vs. 7.5 ml) shown as non-normalized (A) and min-max-normalized (A') data. **(B-B')** Effect of fill volume (fully filled versus partially filled tubes) shown as non-normalized (B) and min-max-normalized (B') data. **(C-C')** Effect of EDTA-plasma tube type as pairwise differential fingerprints: S-K3E (Sarstedt K3-EDTA, 1,6 mg/ml), G-K2E (Greiner K2-EDTA, 1,8 mg/ml), and G-K3E (Greiner K3-EDTA, 1,8 mg/ml) shown as non-normalized (C) and min-max-normalized (C') data. Shaded gray areas indicate the standard deviation of the between-person variability.

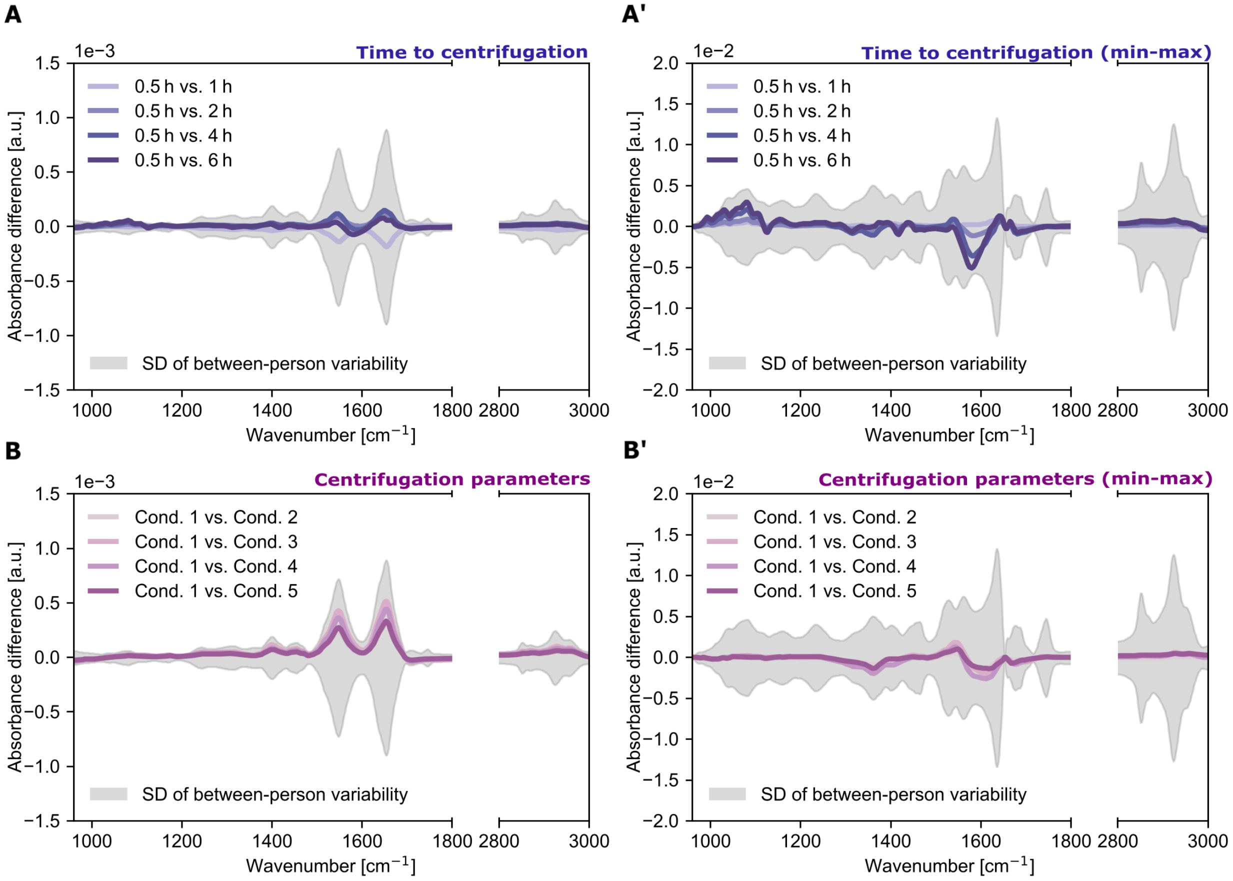

Figure S4: Effects of precentrifugation delays and centrifugation parameters on IMF of EDTA plasma. **(A-A', B-B')** Differential infrared molecular fingerprints (absorbance difference [a.u.]) relative to the reference (0.5 h-delay for (A-A') and 2000 g * 10 min for (B-B')). **(A-A')** Impact of precentrifugation delay (time to centrifugation) shown as non-normalized (A) and min-max-normalized (A') data. **(B)** Impact of centrifugation parameters shown as non-normalized (B) and min-max-normalized (B') data. Centrifugation parameters (relative centrifugal force and duration) for condition 1-5 (Cond. 1-5) can be retrieved from Tab. S2. Shaded gray areas indicate the standard deviation of the between-person variability.

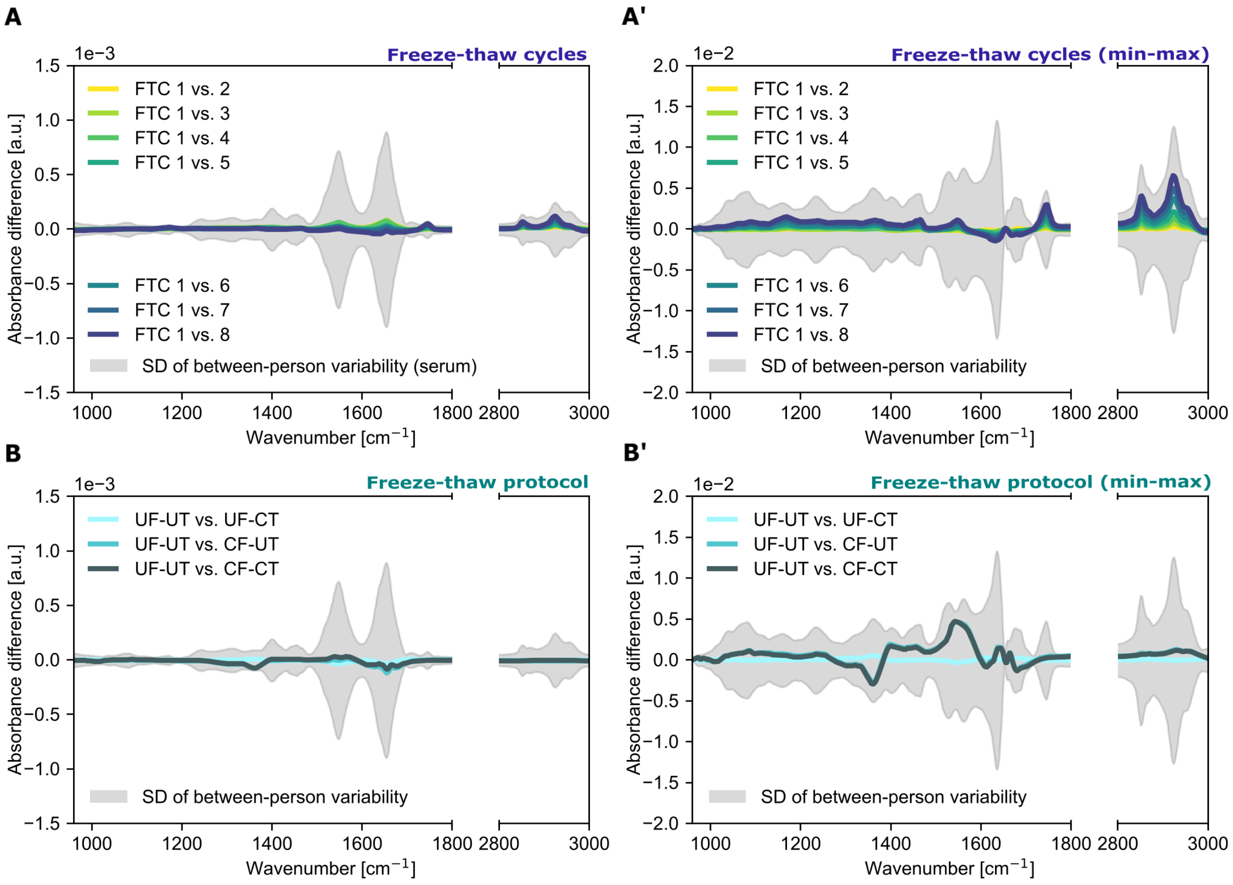

Figure S5: Effects of freeze-thaw cycling and different freezing and thawing procedures on IMF of EDTA plasma. **(A-A', B-B')** Differential fingerprints (absorbance difference [a.u.]) relative to the reference (one freeze-thaw cycle (FTC) for (A-A') and uncontrolled freezing and thawing (UF-UT) after two FTCs for (B-B')) comparing reference workflow samples with samples subjected to repeated freeze–thaw cycles or different, controlled freezing/thawing (CF, CT) workflows. **(A-A')** Impact of different numbers of freeze-thaw cycles on IMF, shown as non-normalized (A) and min-max-normalized (A') data. **(B-B')** Impact of different freezing and thawing protocols on IMF, shown as non-normalized (B) and min-max-normalized (B') data. Shaded gray areas indicate the standard deviation of the between-person variability.

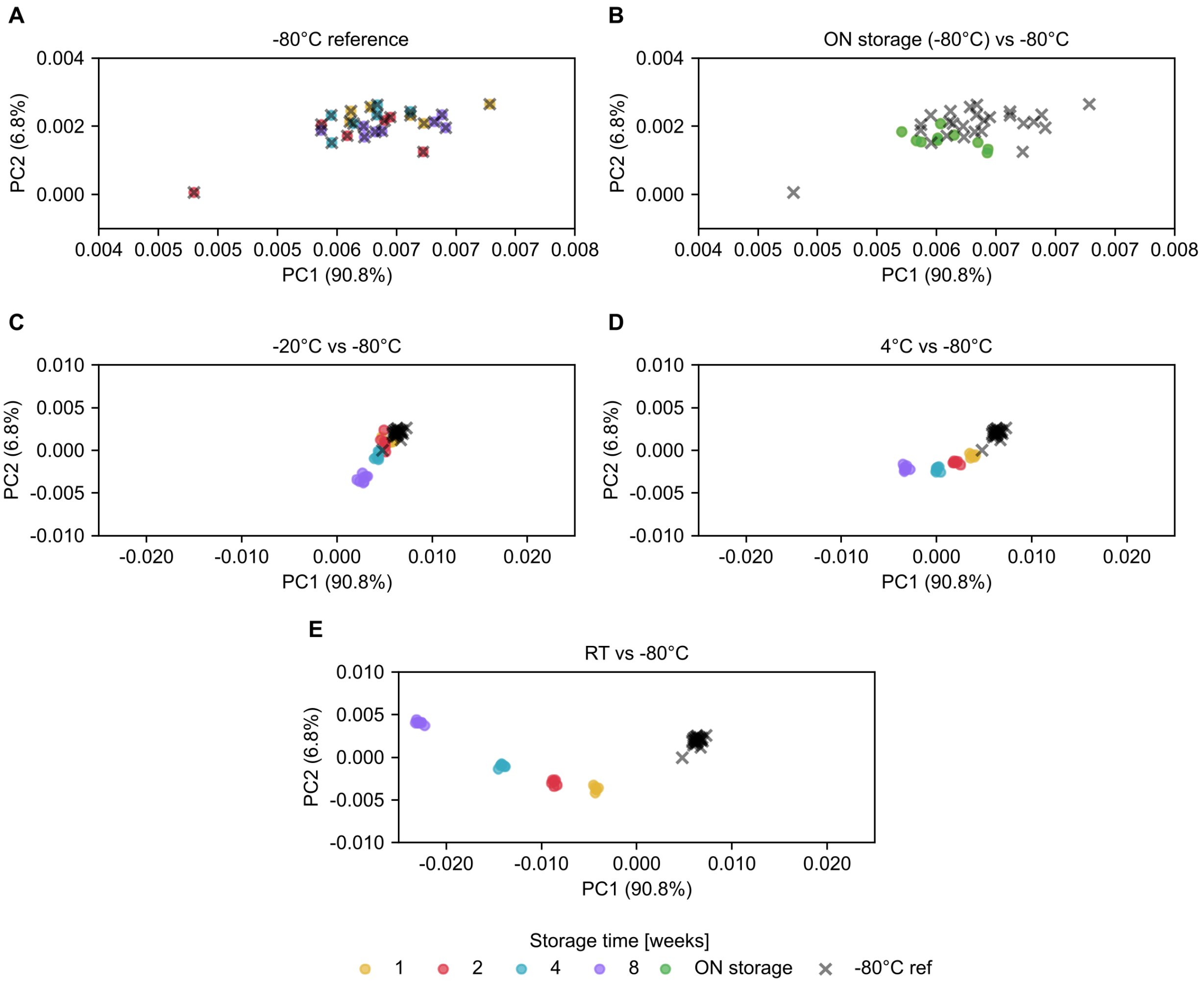

Figure S6: Effect of storage conditions on IR spectra of pooled blood serum (QC serum). Axes represent the first two principal components (PC1 and PC2), explaining the majority of variance in the dataset. Separation between clusters reflects storage-induced spectral changes over time (weeks). Marker color indicates different storage durations. gray crosses denote the $-80\,^{\circ}\mathrm{C}$ reference samples. **(A–E)** Principal component analysis (PCA) of FTIR spectra illustrating the impact of storage temperature depending on storage duration. The reference condition ($-80\,^{\circ}\mathrm{C}$, **A**) is compared to overnight (ON) storage at $-80\,^{\circ}\mathrm{C}$ directly after aliquoting (**B**), and to samples stored at $-20\,^{\circ}\mathrm{C}$ (**C**), $4\,^{\circ}\mathrm{C}$ (**D**), and room temperature (RT, **E**).

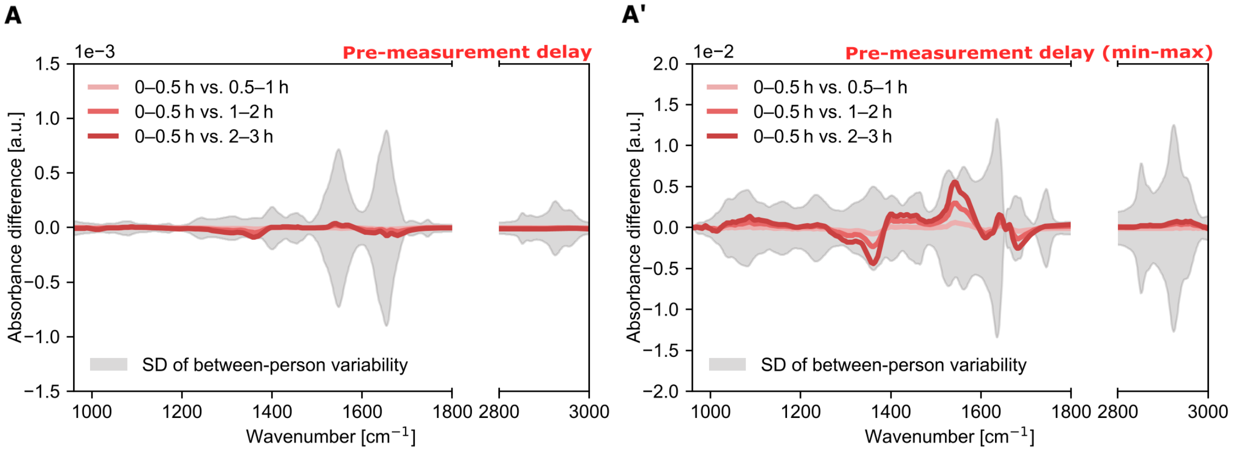

Figure S7: Effects of premeasurement delay time on IMF of pooled blood serum (QC serum) samples. **(A-A')** Differential fingerprints (absorbance difference [a.u.]) between the mean of reference samples (0-0.5 h premeasurement delay) and those subjected to prolonged premeasurement delays, shown as non-normalized (A) and min-max-normalized (A') data. Shaded gray areas indicate the standard deviation of the between-person variability.
